# Necessity and Sufficiency of Ldb1 in the Generation, Differentiation and Maintenance of Non-photoreceptor Cell Types During Retinal Development

**DOI:** 10.3389/fnmol.2018.00271

**Published:** 2018-08-06

**Authors:** Dongchang Xiao, Kangxin Jin, Mengqing Xiang

**Affiliations:** ^1^State Key Laboratory of Ophthalmology, Zhongshan Ophthalmic Center, Sun Yat-sen University, Guangzhou, China; ^2^Guangdong Provincial Key Laboratory of Brain Function and Disease, Zhongshan School of Medicine, Sun Yat-sen University, Guangzhou, China

**Keywords:** Ldb1, LIM-homeodomain, retinal development, transcription factor, retinal progenitor, differentiation, apoptosis

## Abstract

During mammalian retinal development, the multipotent progenitors differentiate into all classes of retinal cells under the delicate control of transcriptional factors. The deficiency of a transcription cofactor, the LIM-domain binding protein Ldb1, has been shown to cause proliferation and developmental defects in multiple tissues including cardiovascular, hematopoietic, and nervous systems; however, it remains unclear whether and how it regulates retinal development. By expression profiling, RNA *in situ* hybridization and immunostaining, here we show that Ldb1 is expressed in the progenitors during early retinal development, but later its expression gradually shifts to non-photoreceptor cell types including bipolar, amacrine, horizontal, ganglion, and Müller glial cells. Retina-specific ablation of *Ldb1* in mice resulted in microphthalmia, optic nerve hypoplasia, retinal thinning and detachment, and profound vision impairment as determined by electroretinography. In the mutant retina, there was precocious differentiation of amacrine and horizontal cells, indicating a requirement of Ldb1 in maintaining the retinal progenitor pool. Additionally, all non-photoreceptor cell types were greatly reduced which appeared to be caused by a generation defect and/or retinal degeneration via excessive cell apoptosis. Furthermore, we showed that misexpressed Ldb1 was sufficient to promote the generation of bipolar, amacrine, horizontal, ganglion, and Müller glial cells at the expense of photoreceptors. Together, these results demonstrate that Ldb1 is not only necessary but also sufficient for the development and/or maintenance of non-photoreceptor cell types, and implicate that the pleiotropic functions of Ldb1 during retinal development are context-dependent and determined by its interaction with diverse LIM-HD (LIM-homeodomain) and LMO (LIM domain-only) binding protein partners.

## Introduction

The interaction between LMO (LIM domain-only) and LIM-HD (LIM-homeodomain) proteins and their binding cofactors, LDB (LIM domain-binding) proteins, has emerged as one of the key mechanisms regulating cell differentiation and tissue development in many organisms including human. There are seven members, Lmo1-7, in the LMO family, of which Lmo1-4 are well studied (Sang et al., 2014). The LIM-HD proteins consist of several families, including Lhx1-9, Isl1-3, Lmx1a, Lmx1b, etc. (Matthews et al., 2008). There are two major members in the LDB family, Ldb1 and Ldb2, that have partially overlapping and redundant functions during embryogenesis and maturation (Matthews and Visvader, 2003; Matthews et al., 2008; Leone et al., 2017). Ldb1 is universally expressed in many tissues and cell types whereas Ldb2 expression is more regionalized. The LIM domains of LMO and LIM-HD proteins compete to bind to the LID (LIM interaction domain) domain of LDB proteins and form complexes with diverse affinities in a dose-dependent manner (Matthews and Visvader, 2003; Matthews et al., 2008). Depending on the spatiotemporally dynamic expression of LMO and LIM-HD factors, the composition of LDB complexes is certainly diverged within tissues and cell types, and apparently dictates their transcriptional activity.

Not surprisingly, as the key component of LDB complexes, Ldb1 exerts myriad functions during development. Given its ubiquitous expression pattern, targeted mutation of *Ldb1* in the mouse caused developmental defects in multiple systems including cardiovascular, craniofacial, digestive/alimentary, growth/size, hematopoietic, mortality/aging, nervous system, reproductive system, renal system and more (Mukhopadhyay et al., 2003; Suleiman et al., 2007; Zhao et al., 2007; Mylona et al., 2013). During cardiogenesis, Ldb1 binds to the key regulator of cardiac progenitors, Isl1, and maintains its stability. The Ldb1/Isl1 complex then orchestrates the cardiac-specific transcription programs (Caputo et al., 2015). Neural crest-specific deletion of *Ldb1* leads to craniofacial defects (Almaidhan et al., 2014), probably mediated by the Ldb1/Lmo4 complex due to its requirement in the neural crest as shown in the zebrafish (Ochoa et al., 2012). In erythropoiesis, Ldb1, Lmo2, Gata-1 and Tal1 form a multi-protein complex as the master regulator to coordinate the erythroid transcription programs (Wadman et al., 1997; Li et al., 2010, 2013; Soler et al., 2010; Love et al., 2014; Stadhouders et al., 2015; Lee et al., 2017). Mutations in the Ldb1 cofactor gene *Lmx1b* causes nail-patella syndrome (Doucet-Beaupre et al., 2015), whose symptoms comprise part of the phenotypes found in *Ldb1* mutants.

During nervous system development, Ldb1 also displays pleiotropic effects in various tissues. Ldb1 with cofactor Lhx1 and Lhx5 are expressed in the Purkinje cells in the developing cerebellum. Compound mutants of *Lhx1* and *Lhx5*, or inactivation of *Ldb1*, resulted in a severe loss of Purkinje cells and demonstrated that the Ldb1 complex is essential for controlling the Purkinje cell fate (Zhao et al., 2007). In the developing cerebral cortex, Ldb1, Lmo4 and Ngn2 align into a complex that drives the acquisition of cortical neuronal identities by activating Ngn2-dependent gene expression (Asprer et al., 2011). In neural tube development, Isl1 competes with Lhx3 and Lhx4 to bind Ldb1, which determines motor neuron versus interneuron binary cell fates (Sharma et al., 1998; Thaler et al., 2002). In the pituitary, mutations in *Lhx3* and *Lhx4* are also the causes for combined pituitary hormone deficiency (CPHD) (Sheng et al., 1996; Netchine et al., 2000; Dateki et al., 2010), indicating that Ldb1/Lhx3/Lhx4 complex is indispensable for pituitary development. In the developing telencephalon, Ldb1 may coordinate with Lhx6 and Lhx8 to regulate differentiation of GABAergic and cholinergic neurons (Zhao et al., 2014). In the midbrain, *Ldb1* deficiency severely reduces its size and causes a loss of dopaminergic neurons, identical to the midbrain phenotype observed in *Lmx1b* mutants (Kim et al., 2016). These findings have demonstrated that Ldb1, depending on its binding cofactors, has many diverse functions in the developing nervous system.

The retina, considered as the most important sensory organ and a part of CNS (central nervous system), has proven to be one of the best models in which to study neural development. The mouse retina is a laminated structure with three layers of cells, the rod and cone photoreceptors in the outer nuclear layer (ONL), the horizontal, amacrine, bipolar and Müller cells in the inner nuclear layer (INL), and retinal ganglion cells and displaced amacrine cells in the ganglion cell layer (GCL) (Masland, 2012; Xiang, 2013; Cepko, 2014; Jin, 2017; Jin and Xiang, 2017). The LDB cofactors have been reported to play crucial roles in retinal development. Lhx2 is an essential organizer of early retinogenesis and participates in RPC (retinal progenitor cell) proliferation. Thus, *Lhx2* inactivation causes a great reduction of RPC population and increases neurogenesis correspondingly (Porter et al., 1997; Gordon et al., 2013). Lhx2 is also essential for retinal gliogenesis, partly by regulating molecules in the Notch pathway (de Melo et al., 2016). Lhx1 and Lhx5 are shown to be required for development of the optic vesicle (Inoue et al., 2013). Lhx1 also determines the terminal differentiation and migration of horizontal cells (Poche et al., 2007). Lhx9, on the other hand, is only required for a very small subset of amacrine cells, the neuronal nitric oxide synthase (nNOS/bNOS/NOS1)-expressing amacrine cells (Balasubramanian et al., 2018). Isl1 is also an important LIM-HD factor expressed in the retina and controls the development of ganglion, bipolar and cholinergic amacrine cells (Elshatory et al., 2007; Mu et al., 2008; Pan et al., 2008). Lmo4 and other LMO members have been demonstrated to be both necessary and sufficient for multiple retinal cell type development (Duquette et al., 2010; Jin et al., 2016). These phenotypes together suggest that Ldb1 and/or Ldb2 may be indispensable in retinal development. Thus, in this study, we systematically investigated the function of Ldb1 during retinal neurogenesis and differentiation by loss-of-function, gain-of-function, RNA-seq and other approaches.

## Materials and Methods

### Animals

All the procedures in animals were carried out according to the IACUC standards, and approved by Zhongshan Ophthalmic Center, Sun Yat-sen University and Rutgers, the State University of New Jersey. All mice were maintained and bred in the Zhongshan Ophthalmic Center Laboratory Animal Center or in the Sun Yat-sen University Laboratory Animal Center. The C57BL6/J mice were purchased from the Vital River Laboratories (Beijing, China). The CD1 mice were obtained from the Charles River Laboratories (Wilmington, MA, United States). The floxed *Ldb1* mouse line was reported previously (Zhao et al., 2007) and maintained by breeding with C57BL6/J mice. It was bred with the Six3-Cre transgenic line (Furuta et al., 2000) to delete *Ldb1* in the developing retina to obtain the *Ldb1*^Δfl/Δfl^ (Six3-Cre;*Ldb1^fl/fl^*) and control (*Ldb1^+/+^*, *Ldb1^+/fl^* and *Ldb1^fl/fl^*) animals. The starting stage of mouse embryos was defined by taking the morning as E0.5 when the copulation plug was seen. All genotypes were determined by PCR.

### RNA-Seq Analysis

RNA was extracted from P0 control and *Ldb1*^Δfl/Δfl^ retinas using the TRIzol reagent (Life Technologies) according to the manufacturer’s instruction. Ribosomal RNA was depleted prior to RNA-seq library preparation. The prepared libraries were sequenced using an Illumina HiSeq X TEN sequencer (Biomarker Technologies, China). The obtained sequence reads were trimmed and mapped to the mouse reference genome (mm10) using HISAT2 (Kim et al., 2015)^1^. Gene expression and changes were analyzed using Cufflinks^2^. Scatter and volcano plot analyses of gene expression levels were performed using the R software^3^. Gene set enrichment analysis (GSEA) was carried out as described (Subramanian et al., 2005), which was followed by network visualization in Cytoscape using the EnrichmentMap plugin (Cline et al., 2007; Merico et al., 2010).

### RNA *in situ* Hybridization and EdU Labeling

RNA *in situ* hybridization was carried out as described using digoxigenin-labeled antisense riboprobes (Sciavolino et al., 1997). The full-length open reading frame (ORF) of *Ldb1* was used to prepare the hybridization probe, which was amplified by PCR from retinal cDNA and subcloned into the pBluescript KS(-) vector (Agilent Technologies). EdU (5-ethynyl-2′-deoxyuridine) labeling was performed as previously described (Luo et al., 2012) using the Click-iT EdU labeling kit (Invitrogen).

### Antibodies and Immunostaining

Tissue processing and immunostaining were carried out as described previously (Li et al., 2004; Mo et al., 2004). The following primary antibodies were used: rabbit anti-GFP (1:1000, 598, MBL International), goat anti-GFP (1:2000, ab6673, Abcam), chicken anti-GFP (1:2000, ab13970, Abcam), mouse anti-glutamine synthetase (1:1000, mab302, Millipore), mouse anti-Brn3a (1:500, MAB1585, Millipore), goat anti-Brn3b (1:500, sc-6026, Santa Cruz Biotech.), goat anti-Bhlhb5/BETA3 (1:2000, sc-6045, Santa Cruz Biotech.), rabbit anti-calbindin D-28 k (1:3000, CB-38, Swant), goat anti-Chx10 (1:2000, sc-21690, Santa Cruz Biotech.), rabbit anti-GABA (1:1000, a2052, Sigma), mouse anti-GAD65 (1:500, 559931, BD Biosciences), mouse anti-GAD67 (1:500, MAB5406, Millipore), goat anti-GLYT1 (1:2000, AB1770, Millipore), mouse anti-Lhx1 [1:10, 4F2, Developmental Studies Hybridoma Bank (DSHB)], rabbit anti-Pax6 (1:2000, ab2237, Millipore), mouse anti-Pax6 (1:1000, Pax6, DSHB), rabbit anti-recoverin (1:4000, ab5585, Millipore), Mouse anti-syntaxin (1:1000, S0664, Sigma), rabbit anti-Sox9 (1:8000, ab5535, Millipore), rabbit anti-Tfap2a/2b (1:1000, ab11828, Abcam), rabbit anti-GFAP (1:1000, Z0334, DAKO), rabbit anti-protein kinase Cα (1:15000, P4334, Sigma), rabbit anti-Ki67 (1:100, MA5-14520, Thermo Fisher), mouse anti-Isl1 (1:500, 40.2D6-s, DSHB), rabbit anti-Ldb1 (1:1000, ab96799, Abcam), rabbit anti-active Caspase 3 (1:100, 559565, BD Biosciences), rabbit anti-tyrosine hydroxylase (1:1000, ab152, Millipore), rat anti-Lmo4 (1:1000, Sum et al., 2002); goat anti-choline acetyltransferase (ChAT) (1:100, AB144P, Millipore), goat anti-Lhx2 (1:2000, sc-19344, Santa Cruz Biotech.) and rabbit anti-Ebf (1:1000, sc-33552, Santa Cruz Biotech.). Secondary antibodies conjugated with Alexa Fluor 488 or 594 were used (Life Technologies). Images were captured by a confocal microscope system (Zeiss, LSM700). The corresponding retinal cell types and subtypes immunolabeled by specific antibodies are listed in **Supplementary Table S1**.

### Quantitative Real-Time RT-PCR (qRT-PCR)

Total RNA was isolated from P0 control and *Ldb1*^Δfl/Δfl^ mouse retinas using the TRIzol reagent, and cDNA was synthesized using the NEB Reverse Transcription Kit. qRT-PCR was performed with the LightCycler^®^ 96 Real-Time PCR System (Roche) and all reactions were carried out in three independent biological replicates. The primer sequences used for qRT-PCR are listed in **Supplementary Table S2**.

### Plasmid Construction and Virus Infection

The full-length ORF of *Ldb1* was obtained by PCR from mouse retinal cDNA, and subcloned into the MMLV-based replication-incompetent retroviral vector pLZRSΔ-IRES-GFP (Mo et al., 2004). The methods to prepare retroviruses, infect retinas and collect samples were described previously (Jin et al., 2010; Jin and Xiang, 2012). In brief, for *in vivo* infection, 1 μl of Ldb1-GFP or control-GFP viruses with 8 μg/ml polybrene (Sigma) were injected into the subretinal space of P0 CD1 neonatal mice, and retinal samples were collected at P21 for analysis. For *in vitro* infection, E13.5 retinal explants were infected with 20 μl of virus mixture containing Ldb1 viruses or control viruses and polybrene, and the samples were collected after 4.5 days for analysis.

### Electroretinographic Analysis

Electroretinograms (ERGs) were recorded from 1-month-old or 3-month-old *Ldb1*^Δfl/Δfl^ mice and age-matched control mice using the amplifier of the RETI-scan system (Roland Consult, RETI-scan, Germany) at a sampling rate of 2 kHz. All mice were dark-adapted overnight and anesthetized with intraperitoneal injection of chloral hydrate (4.5 μg/g body weight), then they were exposed to full-field scotopic flashes of 1.3 ms duration presented by a Ganzfeld (Roland Consult, Germany). Scotopic recordings were obtained from dark-adapted mice at the following increasing light intensities: 0.003, 0.01, 0.03, 0.1, 0.3, 1.0, 3.0, 10 cd.s/m^2^. Thereafter, photopic recordings were performed following 5 min light adaptation intervals on a bright green background light intensity of 20 cd/m^2^. Six levels of stimuli (0.3, 1.0, 3.0, 10, 30, 100 cd.s/m^2^) were used for photopic recordings. For each condition (scotopic and photopic), 3–10 responses were averaged to the luminance of flash stimuli, with the stimulus interval varying from 2 to 10 s at low intensities to 1 min at intensities above 3.0 cd s/m^2^. Analysis of ERG data was carried out as described previously (Yang et al., 2015; Chen et al., 2016).

### Optic Coherence Tomography (OCT) Analysis

For OCT, the eyes were treated with tropicamide phenylephrine to ensure pupil dilation and mice were further anesthetized with intraperitoneal injection of chloral hydrate (4.5 μg/g body weight). Fundus images were captured using the Spectralis OCT (Heidelberg, Germany) according to a previously published protocol (Puk et al., 2013).

### Quantification and Statistical Analysis

For conditional ablation experiments, at least three retinas were analyzed for each control and *Ldb1*^Δfl/Δfl^ animals. Cell marker counting was obtained from 6 non-overlapping fields in similar retinal regions for each retina. Each field was photographed using the confocal microscope at 200x or 400x magnification. For misexpression experiments, depending on the frequency or ratio of each cell type, hundreds to thousands of GFP^+^ cells in each infected retina were scored. And at least 3 retinas were counted for each individual cell marker.

Statistical analysis was performed using the GraphPad Prism 6.0 and Microsoft Excel computer programs. The results are expressed as mean ± SD for experiments conducted at least in triplicates. Unpaired two-tailed Student’s *t*-test was used to assess differences between two groups, and a value of *P* < 0.05 was considered statistically significant.

## Results

### Pattern of *Ldb1* Expression During Mouse Retinal Development

We previously reported an Affymetrix microarray analysis comparing differentially expressed genes between E14.5 *Ptf1a* wildtype and mutant retinas (Wei et al., 2018). Mining this microarray dataset, we found that the expression of *Ldb1* gene was downregulated by approximately two-fold in the mutant retina (**Supplementary Figure S1A**). RNA-seq analysis of developmental stage-specific retinal transcriptomes (KJ and MX, unpublished) revealed that the expression levels of *Ldb1* gradually dropped by about threefold from E13.5 to P13, and henceforth up to at least 9 months remained nearly constant at this lower level (**Supplementary Figure S1B**), suggesting that *Ldb1* has a dynamic temporal expression pattern in the developing and mature retinas.

**FIGURE 1:**
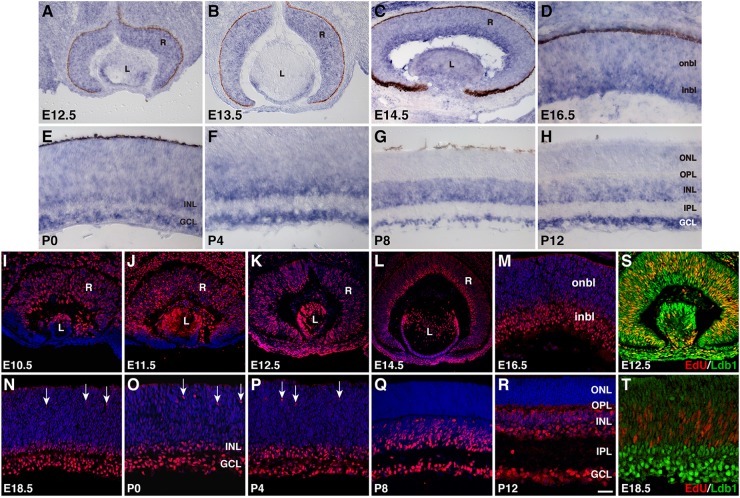
Developmental expression pattern of Ldb1 in the mouse retina. **(A–H)** RNA expression pattern of Ldb1 detected with a Ldb1 antisense probe by RNA *in situ* hybridization. At E12.5 and E13.5, Ldb1 RNA is found in the entire retina. From E14.5 to P4, Ldb1 RNA becomes stronger in differentiated cell layers such as GCL and inbl, but gradually wanes from the onbl and outer layers. From P8 and beyond, Ldb1 RNA is limited in the INL and GCL. **(I–R)** Dynamic expression of Ldb1 protein immunostained with an anti-Ldb1 antibody and counterstained with nuclear DAPI. In agreement with its RNA expression pattern, Ldb1 is expressed in the entire retina before E14.5, then slowly diminished from the onbl and outer layers, and eventually limited to the INL and GCL. **(S,T)** Colabeling of progenitor cells with Ldb1 and EdU in E12.5 and E18.5 EdU-pulse-labeled wildtype retinas. At E12.5, almost all EdU+ cells strongly express Ldb1; however, at E18.5, Ldb1 is weak in EdU cells. GCL, ganglion cell layer; inbl, inner neuroblastic layer; INL, inner nuclear layer; IPL, inner plexiform layer; L, lens; onbl, outer neuroblastic layer; ONL, outer nuclear layer; OPL, outer plexiform layer; R, retina. Arrows in N-P point to migrating horizontal cells. Scale bar: (**L**) 80 μm; (**J,K**) 50 μm; **(A–C,I,M,S)** 40 μm; **(N)** 33.3 μm; **(P,R)** 28.6 μm; **(Q)** 25 μm; **(O)** 22.2 μm; **(M,R,S)** 25 μm; **(D–H,T)** 20 μm.

We investigated further the spatiotemporal expression pattern of *Ldb1* during mouse retinal development by RNA *in situ* hybridization and immunofluorescent staining. At E12.5 and E13.5, a nearly uniform *Ldb1 in situ* hybridization signal was observed in progenitors of the entire retina, which was present also in lens epithelial cells (**Figures 1A,B**). At E14.5 and E16.5, *Ldb1* signal remained in the entire retina but appeared to be stronger within the inner neuroblastic layer (**Figures 1C,D**). At early postnatal stages (P0 to P4), *Ldb1* signal gradually disappeared from the outer neuroblastic layer and became strong in the INL and GCL of the retina (**Figures 1E,F**). From P8 and beyond, it was restricted only to the INL and GCL (**Figures 1G,H**). The observed pattern of *Ldb1 in situ* hybridization signal was similar to that of a previous study which also included stages E14, E16 and P0 (de Melo et al., 2018), except that the signal was incompletely downregulated from the ONL of late postnatal retinas in the previous work whereas we saw a near complete downregulation (**Figures 1G,H**). Ldb1 protein displays an expression pattern quite similar to that of its RNA. From E10.5 to E14.5, it is more or less uniformly expressed in almost all retinal and lens progenitor cells (**Figures 1I–K**). At E14.5 and E16.5, its expression remained strong in the inner neuroblastic layer but became weak in the outer neuroblastic layer (**Figures 1L,M**). From E18.5 to P4, strong Ldb1-immunoreactivity was seen in the INL, GCL, and in some migrating horizontal cells in the outer neuroblastic layer (**Figures 1N–P**). At P8 and P12, Ldb1 was completely downregulated from the ONL and appeared to be expressed in most cells in the INL and GCL (**Figures 1Q,R**). This pattern of retinal Ldb1 immunoreactivity was essentially identical to that of a previous report which also included stages E12.5, E14.5, E16.5, and P0 (Gueta et al., 2016). In E12.5 retinas pulse-labeled with EdU, all EdU-labeled retinal progenitors were immunoreactive for Ldb1; similarly, EdU-labeled retinal progenitors were weakly immunoreactive for Ldb1 in the outer neuroblastic layer in E18.5 retinas pulse-labeled with EdU (**Figures 1S,T**). Thus, Ldb1 is transiently expressed in perhaps all progenitors and then permanently expressed in most non-photoreceptor cells during mouse retinal development.

### Cell Type and Subtype Localization of Ldb1 in the Mature Retina

**FIGURE 2:**
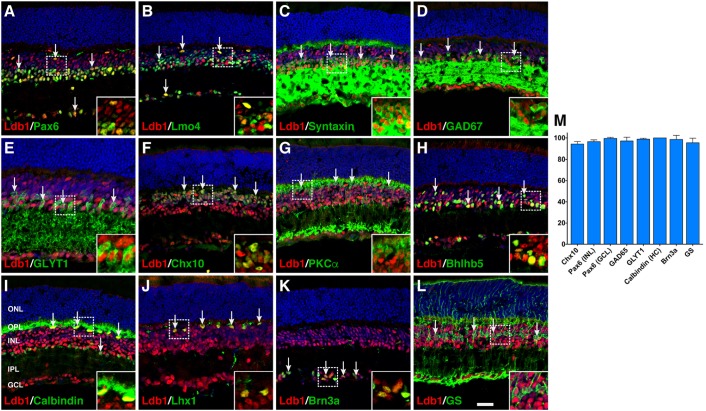
Co-expression of Ldb1 and cell-type markers in P21 wildtype retinas. **(A)** Pax6 and Ldb1 are co-labeled in horizontal and amacrine cells as well as in cells within the GCL. **(B)** Many but not all Ldb1^+^ cells are co-labeled with Lmo4, and vice versa. **(C–E)** Ldb1 immunoreactivity is seen in the great majority of syntaxin^+^ amacrine cells, GAD67^+^ GABAergic amacrine subtypes, and GLYT1^+^ glycinergic amacrine subtypes. **(F,G)** Ldb1 immunoreactivity is seen in the great majority of Chx10^+^ bipolar cells and PKCα^+^ rod bipolar subtypes. **(H)** Ldb1 immunoreactivity is seen in the Bhlhb5^+^ type 2 OFF-cone bipolar cells and GABAergic amacrine cells. **(I,J)** All calbindin^+^ or Lhx1^+^ horizontal cells are co-labeled with Ldb1. **(K)** Ldb1 is expressed in all Brn3a^+^ ganglion cells. **(L)** Ldb1 and glutamine synthetase (GS) are co-expressed in most Müller cells. **(M)** Percentages of marker-positive retinal cells that are immunoreactive for Ldb1. Each histogram represents the mean ± SD for three retinas. All retinal sections were counterstained with nuclear DAPI. Arrows point to representative co-labeled cells. Inset pictures in the lower right corners are magnified from corresponding outlined regions. GCL, ganglion cell layer; HC, horizontal cell; INL, inner nuclear layer; IPL, inner plexiform layer; ONL, outer nuclear layer; OPL, outer plexiform layer. Scale bar: **(A–L)** 28.6 μm.

To investigate its cell type and subtype localization, we co-stained Ldb1 and retinal cell type-specific markers with antibodies in P21 wildtype retinas. The staining of Ldb1 was limited to the INL and GCL where bipolar, amacrine, horizontal, Müller, and ganglion cells reside. We observed extensive colocalization between Ldb1 and Pax6 or Lmo4 in horizontal, amacrine and ganglion cells (**Figures 2A,B**). Consistent with this, Ldb1 was expressed in nearly all amacrine cells immunoreactive for syntaxin, GAD67, GLYT1, or Bhlhb5 (**Figures 2C–E,H**). Similarly, it was co-expressed with calbindin and Lhx1 in all horizontal cells (**Figures 2I,J**). In addition, there was extensive colocalization between Ldb1 and Chx10, PKCα or Bhlhb5 in bipolar cells, between Ldb1 and Brn3a in ganglion cells, and between Ldb1 and glutamine synthetase (GS) in Müller cells (**Figures 2F–H,K,L**). Indeed, quantification of colocalized cells revealed that Ldb1 was expressed in over 90% (94–100%) of cells immunoreactive for Chx10, Pax6, GAD65, GLYT1, calbindin, Brn3a, or GS (**Figure 2M**), suggesting that Ldb1 is expressed in most or all non-photoreceptor cells including bipolar, amacrine, horizontal, ganglion and Müller cells in the mature mouse retina.

### *Ldb1* Inactivation Causes Microphthalmia, Blindness, Optic Nerve Hypoplasia, and Retinal Detachment

**FIGURE 3:**
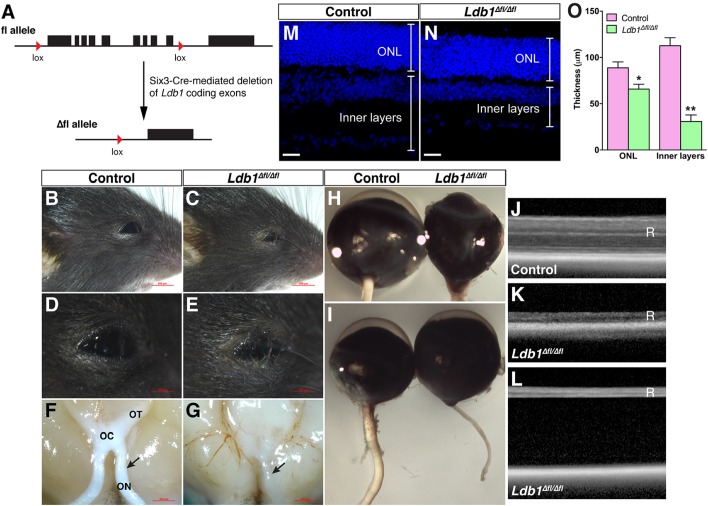
Conditional knockout of *Ldb1* causes defective eye development. **(A)** The cartoon illustrates that Six3-Cre-mediated recombination removes most of *the Ldb1* coding exons. **(B–E)** Loss of *Ldb1* often results in a microphthalmia phenotype. **(F,G)** Atrophic optic nerve, optic chiasm and optic tract in *Ldb1*^Δfl/Δfl^ animals. **(H,I)** Optic nerve hypoplasia and incomplete penetrant microphthalmia phenotype in *Ldb1*^Δfl/Δfl^ mice. **(J,K)** OCT images show that the retina gets much thinner in the mutant mouse. **(L)** OCT image showing that the retina gets detached from the pigment epithelium layer in a severely affected mutant mouse. **(M–O)** The thickness of the ONL and inner layers of wildtype and *Ldb1* mutant retinas, visualized by nuclear DAPI labeling, was quantified at P21. Each histogram in O represents the mean ± SD for 3 animals. ^∗^*p* < 0.01; ^∗∗^*p* < 0.0005. OC, optic chiasm; ON, optic nerve; ONL, outer nuclear layer; OT, optic tract; R, retina. Scale bar: **(M,N)** 20 μm.

Given the dynamic expression of Ldb1 in retinal progenitors and mature cell types, we investigated its retinal function by conditionally inactivating it in retinal progenitor cells using a floxed *Ldb1* allele and the Six3-Cre driver mouse line (**Figure 3A**). Compared to control animals, *Ldb1*^Δfl/Δfl^ mice exhibit several obvious phenotypes: (1) Microphthalmia is associated with some mutant animals although this phenotype is not completely penetrant (**Figures 3B–E,H,I**); (2) There is a dramatic reduction of the optic nerve size in mutant mice. As a result, the optic chiasm and tracts also become atrophic in the mutant (**Figures 3F,G,I**); (3) OCT (optic coherence tomography) revealed that the mutant retina was greatly decreased in thickness (**Figures 3J,K**). Consistent with this, measurement of P21 control and *Ldb1*^Δfl/Δfl^ retinas showed that the thickness of the inner layers was diminished by approximately 73% in the mutant compared to the control, and that of the ONL was reduced by 26% (**Figures 3M–O**); and (4) As determined by OCT, apart from reduced thickness, the mutant retina often got detached from the rest of the eyecup (**Figure 3L**). Therefore, Ldb1 may be required for the formation and maintenance of the retina.

**FIGURE 4:**
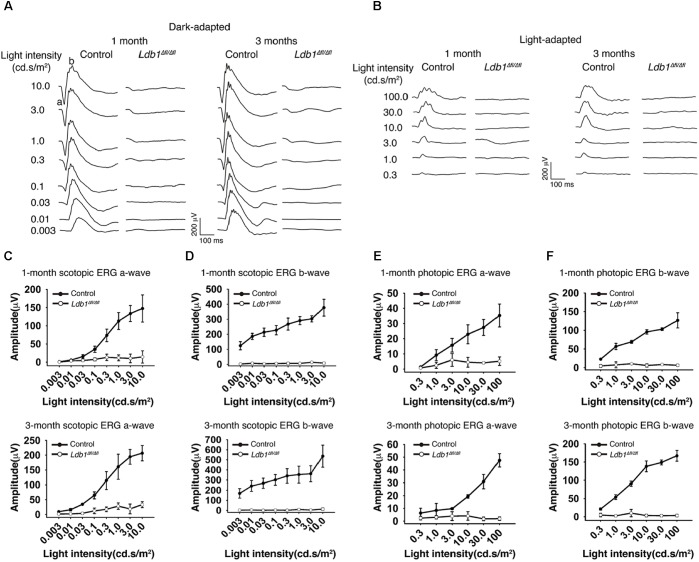
Scotopic and photopic ERG recordings at different flash intensities from control and *Ldb1* mutant mice. **(A,B)** Representative ERG waveforms from dark-adapted **(A)** or light-adapted **(B)** control and mutant animals aged 1 and 3 months. The flash light intensities used to elicit the responses were indicated to the left. **(C,D)** The average amplitudes of scotopic a-waves **(C)** and b-waves **(D)** from control and mutant mice aged 1 and 3 months are shown as a function of light intensities. **(E,F)** The average amplitudes of photopic ERG a-waves **(E)** and b-waves **(F)** from control and mutant mice aged 1 and 3 months are shown as a function of light intensities. Differences between control and mutant animals are statistically significant (*p* < 0.05) in scotopic and photopic examinations at almost all flash intensities except for a couple of weak ones. Values are shown as mean ± SD (*n* = 3) for all tests.

To assess visual function, we recorded ERG responses from 1-month and 3-month old control and *Ldb1*^Δfl/Δfl^ mice. At both ages, under dark- and light-adapted conditions, both a- and b-waves became visible in control animals for flashes at higher intensities. By contrast, neither a- nor b-waves could be seen in mutant mice even for flashes at the highest intensities tested (10.0 cd.s/m^2^ for dark-adapted animals and 100.0 cd.s/m^2^ for light-adapted animals) (**Figures 4A,B**). In agreement with this, the response amplitudes of both a- and b-waves elicited from control animals gradually increased with increasing flash intensities whereas those elicited from *Ldb1*^Δfl/Δfl^ mice remained essentially flat at the baseline (**Figures 4C–F**), indicating that the mutant mice are effectively blind by the age of 1 month, consistent with the observed microphthalmia, retinal thinning and detachment, and optic nerve hypoplasia associated with *Ldb1*^Δfl/Δfl^ mice.

### Severe Loss of Non-photoreceptor Cells in the *Ldb1* Mutant Retina

**FIGURE 5:**
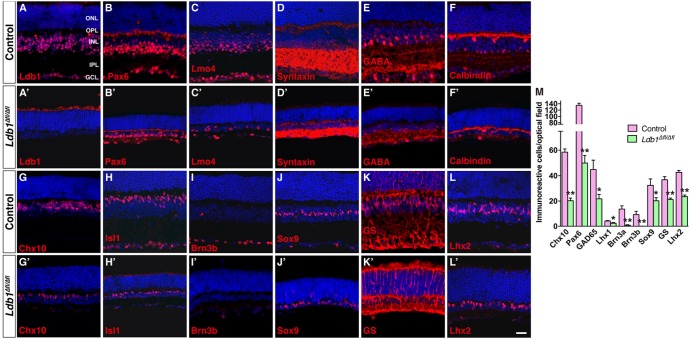
Conditional knockout of *Ldb1* causes loss of all non-photoreceptor cell types. **(A,A’)** Ldb1 immunoreactivity was near completely abolished in the mutant retina. **(B,B’)** Immunoreactivity for Pax6, a cell marker for amacrine, horizontal and ganglion cells, was reduced in the mutant. **(C,C)** The Ldb1 binding cofactor Lmo4, usually present in nearly all major cell types in the INL and GCL, was decreased in the mutant. **(D,D’)** Syntaxin, a marker for all amacrine cells, was dramatically reduced in the mutant. **(E,E’)** GABA immunoreactive amacrine cells were diminished in the mutant. **(F,F’)** Calbindin is expressed in all horizontal cells and some amacrine cells in the wildtype retina. These cells were greatly reduced in the mutant. **(G,G’)** Chx10^+^ bipolar cells were vastly decreased in the mutant. **(H,H’)** Isl1 is present in ON-bipolar, cholinergic amacrine, and ganglion cells. These cells especially ganglion cells were drastically reduced in the mutant. **(I,I’)** Brn3b^+^ ganglion cells nearly disappeared in *the Ldb1* mutant. **(J–L,J’–L’)** Müller cells immunoreactive for Sox9, GS or Lhx2 were decreased in the mutant. **(M)** Quantification of some typical cell markers in control and *Ldb1* mutant retinas. Each histogram represents the mean ± SD for three retinas. ^∗^*p* < 0.05; ^∗∗^*p* < 0.005. All retinal sections were counterstained with nuclear DAPI. GCL, ganglion cell layer; GS, glutamine synthetase; INL, inner nuclear layer; IPL, inner plexiform layer; ONL, outer nuclear layer; OPL, outer plexiform layer. Scale bar: **(A–L,A’–L’)** 20 μm.

The decreased thickness of *Ldb1*^Δfl/Δfl^ retinas likely result from substantial cell loss. We therefore determined by immunostaining the types and subtypes of cells lost in the mutant retina using a battery of cell type-specific antibodies. Immunolabeling of P21 control and mutant retinas with an anti-Ldb1 antibody confirmed near complete absence of Ldb1 expression in *Ldb1*^Δfl/Δfl^ retinas (**Figures 5A,A’**). In the control retina, an anti-Lmo4 antibody labeled most cells in the INL and GCL including bipolar, amacrine, horizontal and ganglion cells and an anti-Pax6 antibody stained all amacrine, horizontal and ganglion cells in the INL and GCL (**Figures 5B,C**). In the mutant, however, there was a great decrease in the abundance of Lmo4- and Pax6-immunoreactive cells (**Figures 5B’,C’**). Consistent with this, there was a dramatic loss in the expression of general amacrine cell markers as well as subtype-specific amacrine cell markers in the mutant retina. These included general amacrine cell markers syntaxin and calbindin, GABAergic amacrine cell markers GABA, GAD65 and GAD67, glycinergic amacrine cell marker GLYT1, cholinergic amacrine cell marker ChAT, and dopaminergic amacrine cell marker TH (**Figures 5D–F,D’–F’** and **Supplementary Figures S2C–G,C’–G’**). Similarly, there were only few horizontal cells immunoreactive for calbindin and Lhx1, and ganglion cells immunoreactive for Brn3a and Brn3b present in *Ldb1*^Δfl/Δfl^ retinas (**Figures 5F,F’,I,I’** and **Supplementary Figures S2H,I,H’,I’**). *Ldb1* ablation also resulted in a sharp reduction in the number of Chx10-immunoreactive bipolar cells and PKCα-immunoreactive rod bipolar cells (**Figures 5G,G’** and **Supplementary Figures S2A,A’**). Additionally, it led to a big loss of cells immunoreactive for Isl1, a marker for bipolar, ganglion and cholinergic amacrine cells, and great loss of neurons immunoreactive for Bhlhb5, a marker for Type 2 OFF-cone bipolar and GABAergic amacrine cells (**Figures 5H,H’** and **Supplementary Figures S2B,B’**). In contrast, there appeared to be only moderate loss of Müller cells immunoreactive for Sox9, GS and Lhx2, and photoreceptor cells positive for recoverin in *Ldb1*^Δfl/Δfl^ retinas (**Figures 5J–L,J’–L’** and **Supplementary Figures S2J,J’**). As indicated by increased GFAP-immunoreactivity (**Supplementary Figures S2K,K’**), there was obvious gliosis in the mutant retina.

Quantification of immunoreactive cells revealed that in *Ldb1*^Δfl/Δfl^ retinas, the number of Chx10^+^, Pax6^+^, GAD65^+^, Lhx1^+^, Brn3a^+^, and Brn3b^+^ cells was reduced by 66.4, 62.9, 52.4, 42.9, 94.3, and 99.5%, respectively, compared to the control retina (**Figure 5M**). Similarly, Sox9^+^, GS^+^ and Lhx2^+^ Müller cells were decreased by 37.7, 42.5, and 44.7%, respectively (**Figure 5M**). The drastic loss of Brn3a^+^ and Brn3b^+^ ganglion cells is consistent with the observed optic nerve hypoplasia associated with mutant animals (**Figure 3**). These results thus suggest that Ldb1 may be required for the generation, differentiation and/or maintenance of all non-photoreceptor cell types including bipolar, amacrine, horizontal, ganglion and Müller cells.

### *Ldb1* Ablation Causes Aberrant Generation of Amacrine and Horizontal Cells and Excessive Cell Death

**FIGURE 6:**
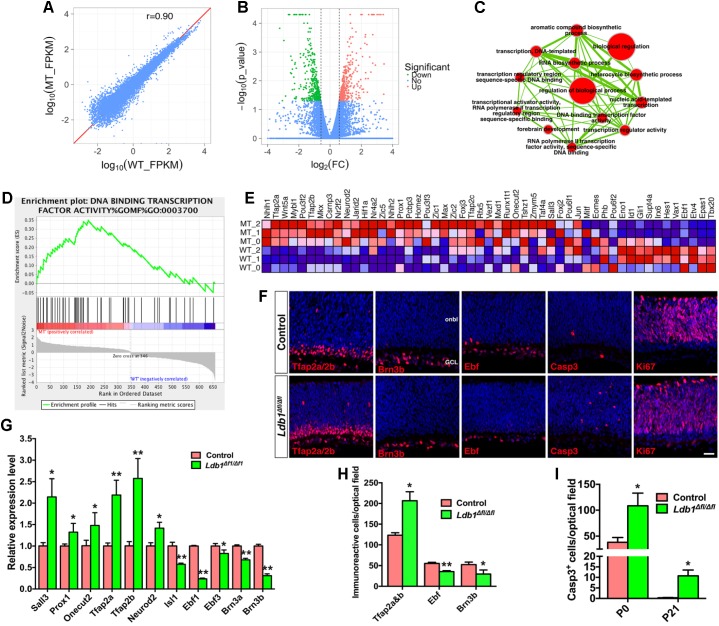
Abnormal cell generation and death in P0 *Ldb1* mutant retinas. **(A)** RNA-seq analysis was carried out to reveal differentially expressed genes in control and *Ldb1* mutant retinas at P0. The scatter plot shows global gene expression profiles in control and mutant retinas. Gene expression levels (FPKM) are depicted in the log_10_ scale. The diagonal line represents equal expression in the two groups. The Pearson correlation coefficient is 0.90. **(B)** Volcano plot (significance vs. fold change) displays significantly downregulated (green) and upregulated (red) genes (fold change ≥ 1.5 and *p* < 0.05) between control and mutant retinas. **(C)** The differentially expressed genes in the *Ldb1* null retina were analyzed for GO term enrichment by gene set enrichment analysis (GSEA). The result was visualized on a network of gene-sets (nodes) connected by their similarity (edges). The node size represents the gene-set size and edge thickness represents the degree of overlap between two gene sets. **(D)** GSEA identified an enriched gene set associated with DNA-binding transcription factor activity. **(E)** GSEA output heatmap of expression levels for the enriched gene set associated with DNA-binding transcription factor activity. **(F)** Sections from P0 control and *Ldb1*^Δfl/Δfl^ retinas were immunostained with the indicated antibodies and counterstained with nuclear DAPI. There is obvious increase of Tfap2a/2b- and activated caspase 3-immunoreactive cells but a decrease of Brn3b- and Ebf-immunoreacitve cells in the mutant retina. Ki67^+^ cells do not appear to change. **(G)** Relative RNA levels of the indicated genes determined by qRT-PCR analysis. Each histogram represents the mean ± SD for triplicate samples. ^∗^*p* < 0.05, ^∗∗^*p* < 0.001. **(H)** Quantitation of cells that are immunoreactive for the indicated markers in P0 control and *Ldb1*^Δfl/Δfl^ retinas. Each histogram represents the mean ± SD for three retinas. ^∗^*p* < 0.05, ^∗∗^*p* < 0.001. **(I)** Quantitation of activated caspase 3^+^ apoptotic cells in control and *Ldb1*^Δfl/Δfl^ retinas at the indicated stages. Each histogram represents the mean ± SD for three retinas. ^∗^*p* < 0.05. Abbreviations: GCL, ganglion cell layer; onbl, outer neuroblastic layer. Scale bar: **(F)** 20 μm.

To understand the molecular basis of Ldb1 regulatory function during retinal development, we carried out RNA-seq analysis to identify genes differentially expressed in *Ldb1* mutant retinas. RNA was extracted from control and *Ldb1*^Δfl/Δfl^ retinas at P0 when mutant retinas are largely intact. This analysis identified 691 genes whose expression level is altered (downregulated or upregulated) by more than 1.5-fold in the mutant retina (**Figures 6A,B** and **Supplementary Table S3**). We performed gene set enrichment analysis (GSEA) of these changed genes followed by network visualization. A major clustered network of GO (gene ontology) terms emerged for a set of genes upregulated in the mutant retina (**Figure 6C**). These genes are enriched for regulation of biological process, RNA biosynthetic process, forebrain development, and in particular, for transcription regulator activity and DNA binding transcription factor activity.

Among the upregulated transcription factor genes are *Tfap2a, Tfap2b, Tfap2c, Nr4a2, Neurod2, Prox1*, *Sall3, Onecut2* (**Figures 6D,E**), which are involved in the specification and differentiation of amacrine and/or horizontal cells (Dyer et al., 2003; Jiang and Xiang, 2009; Cherry et al., 2011; de Melo et al., 2011; Bassett et al., 2012; Sapkota et al., 2014; Jin et al., 2015), suggesting that there may be supernumerary amacrine and horizontal cells generated from progenitors in *Ldb1*^Δfl/Δfl^ retinas at P0 despite their loss at P21. Consistent with this idea, there was a significant increase of amacrine and horizontal cells immunoreactive for Tfap2a/2b in P0 *Ldb1*^Δfl/Δfl^ retinas (**Figures 6F,H**). qRT-PCR assay also validated upregulation of *Tfap2a, Tfap2b, Neurod2, Prox1*, *Sall3*, and *Onecut2* in the null retina (**Figure 6G**). By contrast, *Ldb1* inactivation led to downregulation of a series of ganglion cell marker genes including *Atoh7, Eomes, Ebf1, Irx6, Gap43*, and *Nell2* (**Figure 6E** and **Supplementary Table S3**). In P0 null retinas, we observed by immunolabeling a significant decrease of ganglion cells positive for Brn3b or Ebf factors (**Figures 6F,H**) and confirmed by qRT-PCR downregulation of ganglion cell marker genes *Brn3a, Brn3b, Isl1, Ebf1*, and *Ebf3* (**Figure 6G**), suggesting a defect in ganglion cell differentiation and/or maintenance in the absence of *Ldb1*. In addition, RNA-seq analysis revealed in the P0 null retina downregulation of *Hes1* and *Gli1* (**Figure 6E**), target genes for Notch and Hedgehog signaling, respectively, suggesting a mechanism by which Ldb1 participates in RPC proliferation and maintenance.

In spite of the overproduction of amacrine and horizontal cells in P0 *Ldb1*^Δfl/Δfl^ retinas, both cell types were substantially reduced in the null retina at P21 (**Figure 5**). Similarly, ganglion cell loss was moderate at P0 but reached more than 90% at P21 in the null retina (**Figures 5K**, **6H**). These results suggest a likely ongoing retinal degeneration in the postnatal mutant retina. In agreement, there was a dramatic increase of apoptotic cell death in *Ldb1*^Δfl/Δfl^ retinas at P0 and P21 as determined by immunoreactivity of the activated caspase 3 (**Figures 6F,I**). By contrast, there was no significant difference in the number of Ki67^+^ dividing progenitor cells (mean ± SD, control: 1150.0 ± 116.2 cells/optical field, *n* = 3; *Ldb1*^Δfl/Δfl^: 834.4 ± 184.2 cells/optical field, *n* = 3; *p* = 0.066) in P0 control and mutant retinas (**Figure 6F**). Similarly, we did not observe significant difference in EdU-labeled, Chx10-immunoreactive or Pax6-immunoreactive progenitors between control and mutant retinas at E14.5, E16.5 or E18.5 (**Supplementary Figure S3**).

### Ldb1 Misexpression Facilitates the Differentiation of All Non-photoreceptor Cell Types

Given the necessity for Ldb1 in development of several inner retinal cell types, we investigated its sufficiency to promote non-photoreceptor cell differentiation by a gain-of-function approach using a replication-incompetent murine retroviral vector that carries a GFP reporter (**Supplementary Figure S4A**) (Mo et al., 2004). Retinal progenitors were infected at P0 by subretinal injection of Ldb1-GFP or Control-GFP viruses and we analyzed the laminar position and morphology of GFP^+^ cells in infected retinas at P21. In retinas infected with Ldb1-GFP viruses, the fraction of GFP^+^ cells in the ONL dropped from 89.3% in the control retina to 76.8% (**Supplementary Figures S3B–D**). By contrast, the fraction of GFP^+^ cells distributed in the INL more than doubled (from 10.7 to 23.1%) (**Supplementary Figures S4B–D**). Thus, Ldb1 misexpression significantly alters the proportions of progeny distributed in different retinal cell layers.

**FIGURE 7:**
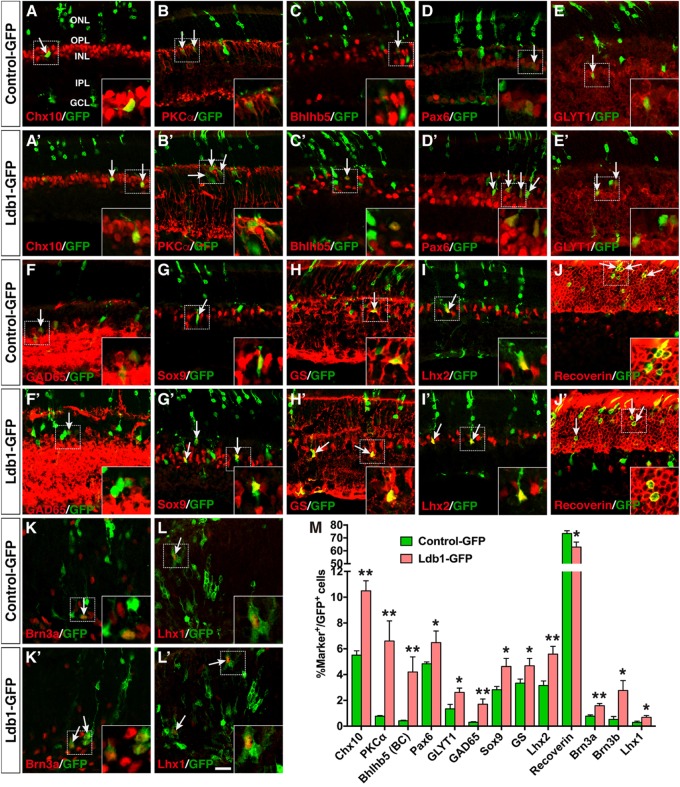
Effects of misexpressed Ldb1 on the differentiation of retinal cell types and subtypes. **(A–J,A’–J’)** Ldb1-GFP or control-GFP retroviruses were injected into the subretinal space at P0 and retinas were collected at P21 for analysis. **(A–C,A’–C’)** Misexpressed Ldb1 promoted the differentiation of Chx10^+^ bipolar cells, PKCα^+^ rod bipolar cells and Bhlhb5^+^ type 2 OFF-cone bipolar cells. **(D–F,D’–F’)** Misexpressed Ldb1 promoted the formation of Pax6^+^ amacrine cells, GLYT1^+^ glycinergic amacrine cells and GAD65^+^ GABAergic amacrine cells. **(G–I,G’–I’)** Misexpressed Ldb1 promoted the differentiation of Müller cells positive for Sox9, GS or Lhx2. **(J,J’)** Misexpressed Ldb1 decreased the number of recoverin^+^ photoreceptors. **(K,L,K’,L’)** E13.5 retinal explants were infected with Ldb1-GFP or control-GFP retroviruses and collected for analysis after 4.5 days in culture. Misexpression of Ldb1 increased Brn3a^+^ ganglion cells and Lhx1^+^ horizontal cells. **(M)** Quantification of GFP^+^ cells that become immunoreactive for a series of cell type-specific markers. Each histogram represents the mean ± SD for three retinas. A range of 400–3696 GFP^+^ cells was scored in each retina depending on the abundance of co-labeled cells. ^∗^*p* < 0.05, ^∗∗^*p* < 0.005. Arrows point to representative colocalized cells and insets show corresponding outlined regions at a higher magnification. BC, bipolar cell; GCL, ganglion cell layer; GS, glutamine synthetase; INL, inner nuclear layer; IPL, inner plexiform layer; ONL, outer nuclear layer; OPL, outer plexiform layer. Scale bar: **(A-L,A’-L’)** 20 μm.

The increased GFP^+^ cells in the INL of retinas infected with Ldb1-GFP viruses could represent more amacrine, horizontal, bipolar, and/or Müller cells. To distinguish these possibilities, we used a variety of cell type-specific markers to analyze the types of GFP^+^ cells and found that forced Ldb1 expression resulted in an increase of several cell types: (1) It significantly increased the percentage of Chx10^+^ bipolar cells from 5.5 to 10.5%, PKCα^+^ rod bipolar cells from 0.8 to 6.6%, and Bhlhb5^+^ type 2 OFF-cone bipolar cells from 0.4 to 4.2% (**Figures 7A–C,A’–C’,M**); (2) It increased the proportion of Pax6^+^ amacrine cells from 4.8 to 6.5%, GLYT1^+^ glycinergic amacrine cells from 1.3 to 2.6%, and Gad65^+^ GABAergic amacrine cells from 0.3 to 1.7% (**Figures 7D–F,D’–F’,M**); 3) The fraction of Sox9-, GS- and Lhx2-immunoreactive Müller cells was also increased from 2.8 to 4.6%, from 3.3 to 4.7%, and from 3.2 to 5.6%, respectively, in retinas infected with Ldb1-GFP viruses (**Figures 7G–I,G’–I’,M**); and (4) In contrast, misexpressed Ldb1 caused a significant decrease of recoverin-immunoreactive photoreceptors from 73.4 to 62.9% (**Figures 7J,J’,M**).

To assess the effect of misexpressed Ldb1 on development of ganglion and horizontal cells, which are born at embryonic stages, we infected E13.5 retinal explants with Ldb1-GFP or Control-GFP viruses, and harvested them after 4.5 days in culture to analyze the differentiation of ganglion and horizontal cells. We found that misexpressed Ldb1 increased Brn3a^+^ ganglion cells from 0.8 to 1.6%, Brn3b^+^ ganglion cells from 0.5 to 2.8%, and Lhx1^+^ horizontal cells from 0.3 to 0.7% (**Figures 7K–M,K’,L’**). Together, these misexpression results thus suggest that Ldb1 is able to promote differentiation of all non-photoreceptor cell types including bipolar, amacrine, horizontal, ganglion, and Müller cells which are distributed in the INL and GCL, while inhibiting differentiation of photoreceptor cells located in the ONL.

## Discussion

### Role of Ldb1 in Retinal Progenitor Cells

**FIGURE 8:**
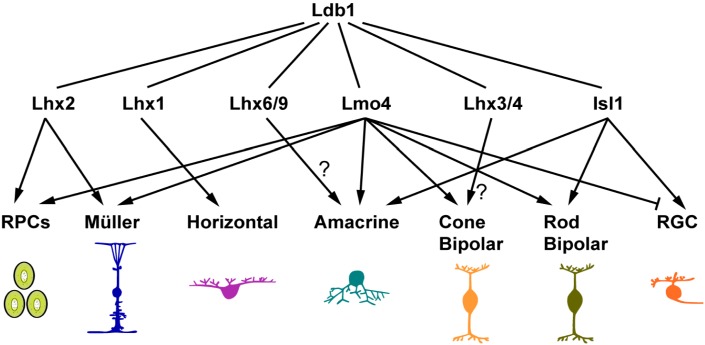
Ldb1 and its cofactors in retinal development and maintenance. The pleiotropic functions of Ldb1 are dependent on its diverse LIM-HD and LMO interacting cofactors. Many known Ldb1 cofactors are expressed in developing and mature retinal cells. Some of them, such as Lhx2 and Lmo4, are involved in cell fate specification. Others do not participate in specification but are required for terminal differentiation and cell survival. For example, Lhx1 does not specify the horizontal cell fate but is required to activate the terminal differentiation program in the precursors, and to control the migration of maturing horizontal cells. The functions of Lhx3/4 in bipolar cells and Lhx6 in amacrine cells are yet to be determined. Nearly all retinal cell types listed here have more than one cofactors co-expressed. How these seemingly competing cofactors coexist and coordinate with Ldb1 function in the same cell remains to be a challenge to unravel.

In the developing retina, Ldb1 expression overlaps with those of LMO and LIM-HD cofactors. In the optic vesicle and early eyecup, Ldb1 is expressed in the RPCs of the entire retina, overlapping with Ldb2 and Lhx2 expression (Gordon et al., 2013; Roy et al., 2013; Balasubramanian et al., 2014; Gueta et al., 2016). Ldb1, Ldb2 together with Lhx2 maintain the early RPC pool by regulating RPC proliferation and differentiation (Gordon et al., 2013; Gueta et al., 2016). Lmo4 and possible other LMO factors are also expressed in the early RPCs (Jin et al., 2016), and compete with Lhx2 for binding to LDBs. The LDB-Lmo4 complex in early RPCs may inhibit differentiation of early retinal neurons (Jin et al., 2016). Therefore the LDB-Lhx2 and LDB-Lmo4 complexes together may prevent the early RPC pool from precocious differentiation into early-born neurons (**Figure 8**). Consistent with this postulation, we have shown in this study that *Ldb1* ablation leads to supernumerary amacrine and horizontal cells at P0 presumably as a result of precocious differentiation of early RPCs.

### Diverse Functions of Ldb1 Dependent on Binding Cofactors During Retinal Development and Maintenance

At stages following RPC generation, Ldb1 gradually appears in postmitotic and differentiating cells (**Figure 1**). Misexpression of Ldb1 is sufficient to specify from progenitors non-photoreceptor cell types including ganglion, bipolar, amacrine, horizontal, and Müller cells, and its inactivation results in loss of all these cell types, demonstrating that Ldb1 in association with various binding partners is both necessary and sufficient for the generation and/or maintenance of non-photoreceptor cell types (**Figure 8**). Isl1 and Lmo4 are known Ldb1 cofactors expressed in mature ganglion cells. During mouse retinogenesis, Isl1 expression is initiated from E11.5 in ganglion cells and Lmo4 is expressed from E16.5. Subsequently both factors are permanently expressed in this cell type (Mu et al., 2008; Pan et al., 2008; Jin et al., 2016). Coincidently, in both *Isl1^Δfl/Δfl^* (Mu et al., 2008; Pan et al., 2008) and *Ldb1*^Δfl/Δfl^ mutant mice, ganglion cells were generated but decreased quickly and apoptotic cell death was shown to increase significantly, indicating that the Ldb1-Isl1 complex may not be required for the specification of ganglion cells but participates in maintaining their survival, consistent with previous reports (Mu et al., 2008; Pan et al., 2008). Failure to maintain ganglion cells then leads to optic nerve hypoplasia in both *Ldb1* and *Isl1* null mice (Mu et al., 2008; Pan et al., 2008) (**Figure 3**). Despite the unnecessity of Isl1 in the specification of ganglion cells, we showed that forced Ldb1 expression was able to substantially increase their number, suggesting that the Ldb1-Isl1 complex may be sufficient to promote ganglion cell differentiation. The Ldb1-Lmo4 complex appears to inhibit ganglion cell generation in embryonic retinal progenitors (Jin et al., 2016) (**Figure 8**) but its function in ganglion cells *per se* is unclear. Even though ganglion cells seemed not to be affected in Lmo4*^Δfl/Δfl^* mice (Duquette et al., 2010; Jin et al., 2016), we cannot rule out the possibility that the Ldb1-Lmo4 complex is also important to sustain ganglion cell survival due to the redundancy of LMO factors in this cell type.

Isl1 and Lmo4 are also expressed in developing and mature bipolar cells, and thus have a role in their differentiation and/or survival (**Figure 8**). Lmo4 is expressed in almost all bipolar cells and we have demonstrated that Lmo4 and other LMO factors are sufficient to promote the bipolar cell fate (Jin et al., 2016), implicating a role for the Ldb1-Lmo4 complex in bipolar cell specification. On the other hand, Isl1 is primarily expressed in rod and ON-cone bipolar cells (Elshatory et al., 2007). The phenotype of its inactivation in the mouse retina suggests that the Ldb1-Isl1 complex is not involved in the specification of bipolar cells but required for their differentiation (Elshatory et al., 2007). Recently, Lhx3 and Lhx4 are identified in mature cone bipolar cells, and thus likely involved in their maintenance (Balasubramanian et al., 2014) (**Figure 8**).

In this study, we have shown that Ldb1 is present in perhaps all developing and mature amacrine, horizontal and Müller cells and required for their differentiation and/or maintenance. We have also demonstrated in this and previous work that its misexpression is sufficient to specify the amacrine and Müller cell fates, and so is Lmo4 misexpression (Jin et al., 2016). These results together thus indicate an important role for the Ldb1-Lmo4 complex in the specification of these two cell types (**Figure 8**). The amacrine cells are a cohort of around 30 subtypes (Masland, 2001). The loss of cholinergic amacrine cells in *Isl1* null retinas suggests a role for the Ldb1-Isl1 complex in specifying this subtype (Elshatory et al., 2007). It is likely that there are additional unidentified Ldb1 cofactors that participate in specifying amacrine subtype identities. In this aspect, a recent knockout study has implicated a role of the Ldb1-Lhx9 complex in the specification of the neuronal nitric oxide synthase-expressing amacrine cells (Balasubramanian et al., 2018) (**Figure 8**). Lhx1 is expressed in horizontal cells and its ablation results in defects in their terminal differentiation and migration (Liu et al., 2000; Poche et al., 2007), suggesting an important function of the Ldb1-Lhx1 complex in horizontal cell development (**Figure 8**). Apart from the essential role in RPC maintenance, the Ldb1-Lhx2 complex is also implicated in the normal development of Müller glial cells as well as in maintaining them in a non-reactive state (de Melo et al., 2012, 2016) (**Figure 8**). In agreement with this, prominent gliosis was found to occur in the *Ldb1*^Δfl/Δfl^ retina.

The loss of non-photoreceptor cells and presumably rapid degeneration in the *Ldb1* mutant retina are most likely caused by a cell-autonomous effect since Ldb1 is expressed in all non-photoreceptor cell types. Nevertheless, this implies that either Ldb2 is not expressed in these cells or unable to completely compensate for the loss of Ldb1 in these cells. Besides non-photoreceptor cells, we also noticed a moderate loss of photoreceptors in the mutant retina, which may result from a collateral effect caused by the loss of INL and GCL cells. For example, genetic ablation of horizontal cells or their developmental loss both lead to secondary degeneration of other retinal cell types including photoreceptors (Sonntag et al., 2012; Keeley et al., 2013; Wu et al., 2013).

### Distinct Features of the Present Study and Novel Insights Gained

While this project was ongoing, two reports studying the LDBs in the retina were published recently (Gueta et al., 2016; de Melo et al., 2018). Despite some similar and consistent discoveries made in our and previous studies, our current work provides a hugely different set of data, gains several novel insights, makes several clarifications, and emphasizes on many new aspects:

(1) In the previous knockout study (Gueta et al., 2016), the majority of the data was collected from the *Ldb1* and *Ldb2* compound knockout mice. Ours were gathered from *Ldb1* single knockout mice, pinpointing the non-redundant function of Ldb1 in the retina; (2) The Cre transgenic driver mouse lines used to perform retina-specific ablation of *Ldb1* are different in our and previous knockout studies. We utilized the Six3-Cre line (Furuta et al., 2000) to inactivate *Ldb1* in the entire retina, whereas the Pax6 α-Cre line was used to inactivate *Ldb1* only in the distal retina in the previous report (Gueta et al., 2016). The Six3-Cre line offers the advantage for us to evaluate the whole-range of retinal defects resulting from *Ldb1* inactivation such as optic nerve hypoplasia and retinal detachment which were unseen in the prior study; (3) We investigated the profound visual function loss in adult *Ldb1* null mice by ERG, an advantage afforded by the Six3-Cre line, which was untouched in the previous work; (4) We demonstrated by a gain-of-function approach the sufficiency of Ldb1 in promoting the differentiation of all non-photoreceptor cell types at the cost of photoreceptors. The previous overexpression study (de Melo et al., 2018) focused on Müller cells and thus barely checked effects on other cell types. Moreover, forced expression of Ldb1 was found to inhibit gliogenesis in the prior study (de Melo et al., 2018); and (5) In contrast to the previous conclusion that Ldb2 is sufficient to substitute Ldb1 in RPCs and Müller cells (Gueta et al., 2016), our data have demonstrated that Ldb1 has unique functions in these two types of cells that cannot be completely compensated for by Ldb2. Several lines of evidence including RNA-seq, qRT-PCR and immunostaining results all indicate that *Ldb1* inactivation causes aberrant generation of supernumerary amacrine and horizontal cells as a result of precocious differentiation of early RPCs. In our work, loss of *Ldb1* function resulted in decreased Müller cells whereas its overexpression promoted their differentiation. Moreover, *Ldb1* ablation also caused prominent gliosis as indicated by elevated GFAP immunoreactivity, suggesting that Ldb1 has a non-redundant role in normal Müller cell development as well as in preventing them from reactivation, much as what Lhx2 does in the retina (de Melo et al., 2012, 2016).

In the previous knockout study (Gueta et al., 2016), Müller cell loss and increased GFAP expression were not observed in retinas of the *Ldb1* single knockout mice. This discrepancy might be due to differences in the observation time window and/or Cre driver mouse line used. For instance, we performed analyses of mutant animals up to P21 and beyond whereas the latest stage analyzed was P14 in the earlier study (Gueta et al., 2016). In addition, no quantification analysis of Müller cells was carried out between the control and *Ldb1* knockout mice in the previous work. If there was only mild Müller cell loss at P14 in the mutant retina, it would be easily missed without careful quantification. In agreement with the earlier knockout study (Gueta et al., 2016), we did not observe significant difference in proliferative RPCs in embryonic and neonatal mutant retinas, implying that Ldb2 may be able to sustain the RPC pool in the absence of Ldb1. However, we found that *Ldb1* ablation led to abnormal generation of amacrine and horizontal cells most likely resulting from precocious differentiation of early RPCs, indicating that Ldb1 may have a unique role in RPCs that cannot be completely compensated for by Ldb2. We gained this insight by benefiting from the RNA-seq and associated bioinformatic analyses which were not performed in the earlier knockout study.

In a recent study focused on exploring the molecular basis of Müller gliogenesis (de Melo et al., 2018), a partial Ldb1 overexpression analysis was conducted, which showed that Ldb1 overexpression reduced Müller cell differentiation but had no effect on amacrine and photoreceptor cells. By contrast, we found that overexpressed Ldb1 was able to promote differentiation of all these three cell types. One explanation for this discrepancy might lie in the different approaches used for overexpression between these two studies, i.e., retroviral- vs. electroporation-mediated overexpression. We used replication-incompetent murine retroviruses to mediate Ldb1 overexpression only in dividing retinal progenitors whereas electroporation-mediated overexpression would allow Ldb1 expression plasmids to enter and express in any kinds of progenitor, differentiating and differentiated cells. As such, the outcome of these two approaches might be different, for instance, the effect of Ldb1 overexpression on Müller cell generation might represent the combined effects on both mitotic and postmitotic cells in electroporation-mediated experiments. In addition, electroporation typically delivers many gene copies to a cell under the control of a very strong CAG promoter whereas retroviral vectors deliver a single gene copy under the control of a moderately strong promoter. Thus, the two overexpression approaches would lead to different expression levels of Ldb1 which might contribute to the different results observed between our and previous studies. In our experience, the electroporation-mediated approach sometimes could mask subtle and moderate differences, which might explain why no significant effect of Ldb1 overexpression was observed on amacrine and photoreceptor cells in the previous study (de Melo et al., 2018).

In summary, using loss-of-function and gain-of-function approaches, we have demonstrated that, besides its role in maintaining the RPC pool, Ldb1 is both necessary and sufficient for the specification of multiple cell types during retinogenesis. In addition, it is continuously expressed in mature non-photoreceptor cells in the INL and GCL, and serves to maintain their survival and perhaps physiological functions. Therefore, we have revealed indispensable roles of Ldb1 in both developing and mature retinas. The pleiotropic functions of Ldb1 appear to be context-dependent and determined by its interaction with diverse LIM-HD and LMO partners.

## Author Contributions

DX, KJ, and MX conceived and designed the research. DX, KJ, and MX performed the experiments. DX, KJ, and MX analyzed the data and wrote the paper.

## Conflict of Interest Statement

The authors declare that the research was conducted in the absence of any commercial or financial relationships that could be construed as a potential conflict of interest.

## Footnotes

1 http://ccb.jhu.edu/software/hisat2/index.shtml

2 http://cole-trapnell-lab.github.io/cufflinks/

3 http://cran.r-project.org
