# Supplementary material for: Necessity and Sufficiency of Ldb1 in the Generation, Differentiation and Maintenance of Non-photoreceptor Cell Types During Retinal Development
Source: Front Mol Neurosci. 2018 Aug 6;11:271. doi: 10.3389/fnmol.2018.00271 (PMC6087769; doi:10.3389/fnmol.2018.00271)
Supplement: Supplementary file 4 [file Data_Sheet_1.PDF]

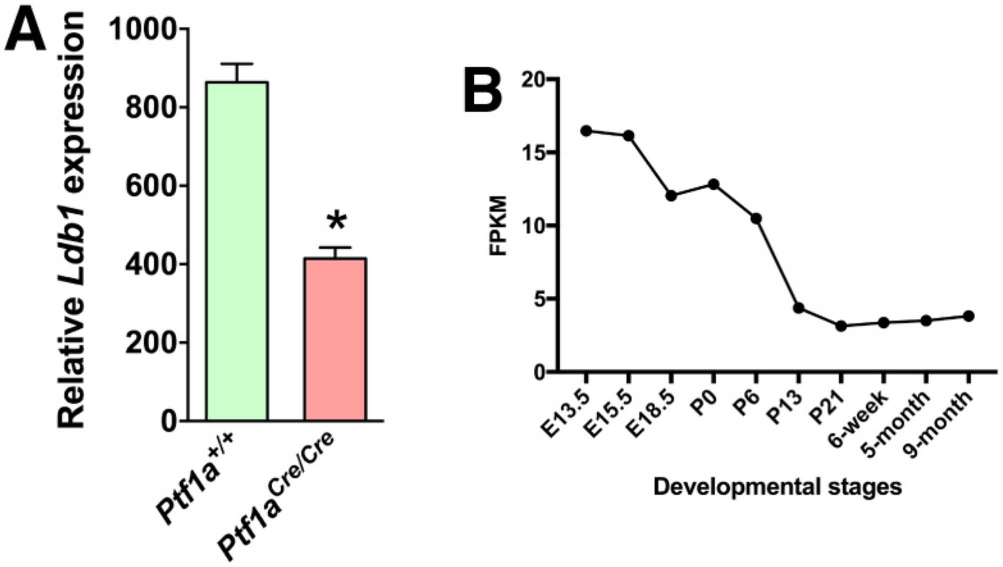

Figure S1 *Ldb1* expression in wildtype and *Ptf1a* mutant retinas. **(A)** Microarray analysis revealed that *Ldb1* expression was significantly downregulated in the *Ptf1a* mutant retina when compared to the wildtype. *p<0.0001. **(B)** Temporal expression levels (FPKM) of *Ldb1* in developing and mature wildtype retinas as determined by RNA-seq analysis.

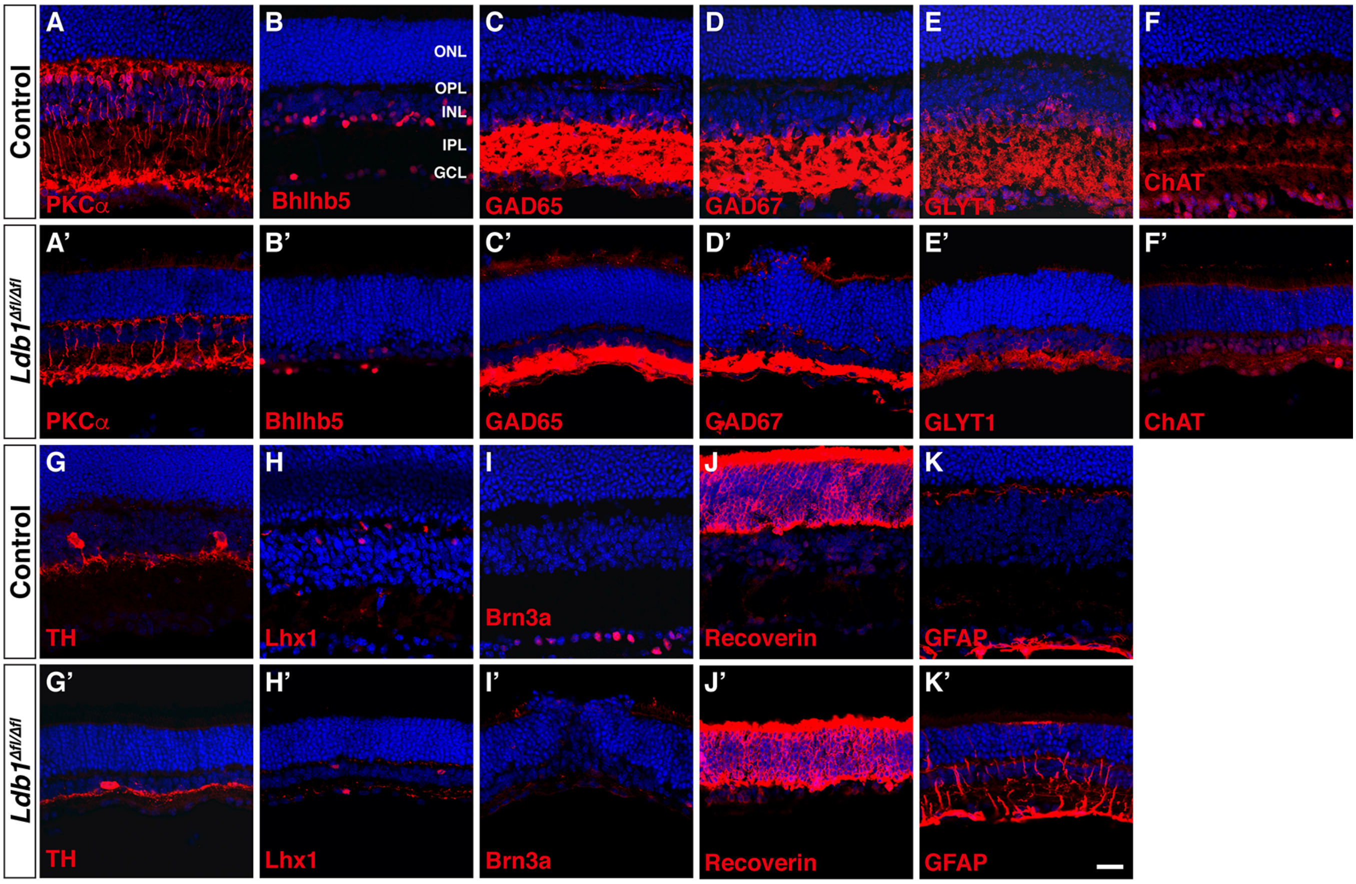

Figure S2 Effect of *Ldb1* inactivation on different retinal cell types. **(A,A')** PKCα$^+$ rod bipolar cells were decreased in the mutant retina. **(B,B')** Bhlhb5$^+$ cone bipolar and GABAergic amacrine cells were reduced in the mutant. **(C-G,C'-G')** The amacrine subtypes including GAD65$^+$ or GAD67$^+$ GABAergic, GLYT1$^+$ glycinergic, ChAT$^+$ cholinergic, and TH$^+$ dopaminergic amacrine cells, were all decreased in the mutant. **(H,I,H',I')** The early-born retinal cell types, such as Lhx1$^+$ horizontal cells and Brn3a$^+$ ganglion cells, were also reduced in P21 mutant retinas. **(J,J')** Recoverin$^+$ photoreceptors were decreased in the mutant retina. **(K,K')** GFAP$^+$ cells were greatly increased in the GCL and INL in the mutant. Abbreviations: GCL, ganglion cell layer; INL, inner nuclear layer; IPL, inner plexiform layer; ONL, outer nuclear layer; and OPL, outer plexiform layer. Scale bar: A-K, A'-K', 20 μm.

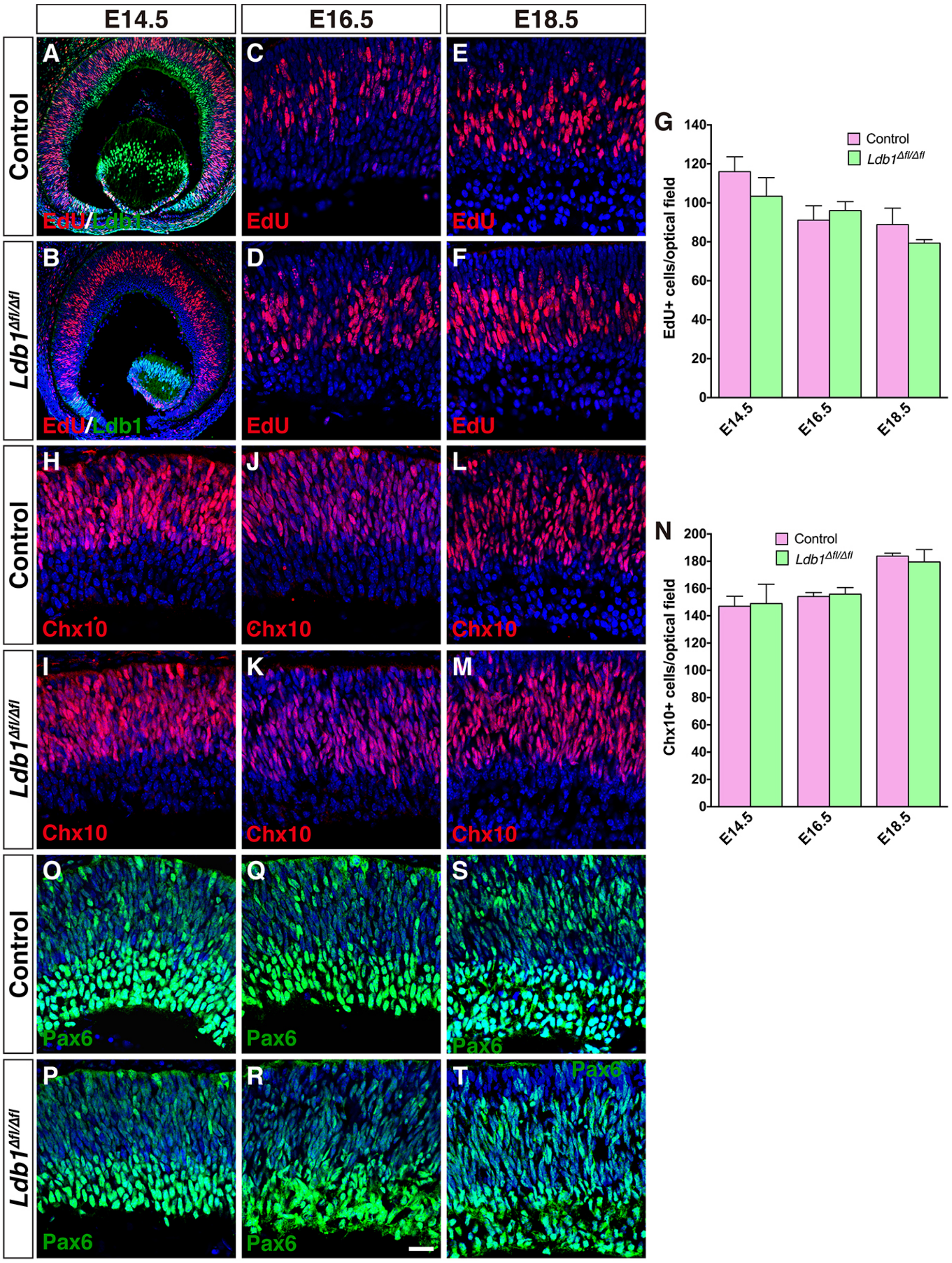

Figure S3 Effect of *Ldb1* inactivation on retinal progenitor cells at embryonic stages. (**A-F**) Retinal sections from E14.5, E16.5 and E18.5 control and *Ldb1*$^{\Delta fl/\Delta fl}$ embryos pulse-labeled by EdU were color-labeled for EdU and/or immunostained with an anti-Ldb1 antibody. (**G**) Quantitation of EdU^{+} dividing cells in control and *Ldb1*$^{\Delta fl/\Delta fl}$ retinas. Each histogram represents the mean±SD for three retinas. (**H-M**) E14.5, E16.5 and E18.5 control and *Ldb1*$^{\Delta fl/\Delta fl}$ retinal sections were immunostained with an anti-Chx10 antibody. (**N**) Quantitation of Chx10^{+} cells in control and *Ldb1*$^{\Delta fl/\Delta fl}$ retinas. Each histogram represents the mean±SD for three retinas. (**O-T**) E14.5, E16.5 and E18.5 control and *Ldb1*$^{\Delta fl/\Delta fl}$ retinal sections were immunostained with an anti-Pax6 antibody. All sections were counterstained with nuclear DAPI. Scale bar: A,B, 40 μm; C-F,H-M,O-T, 20 μm.

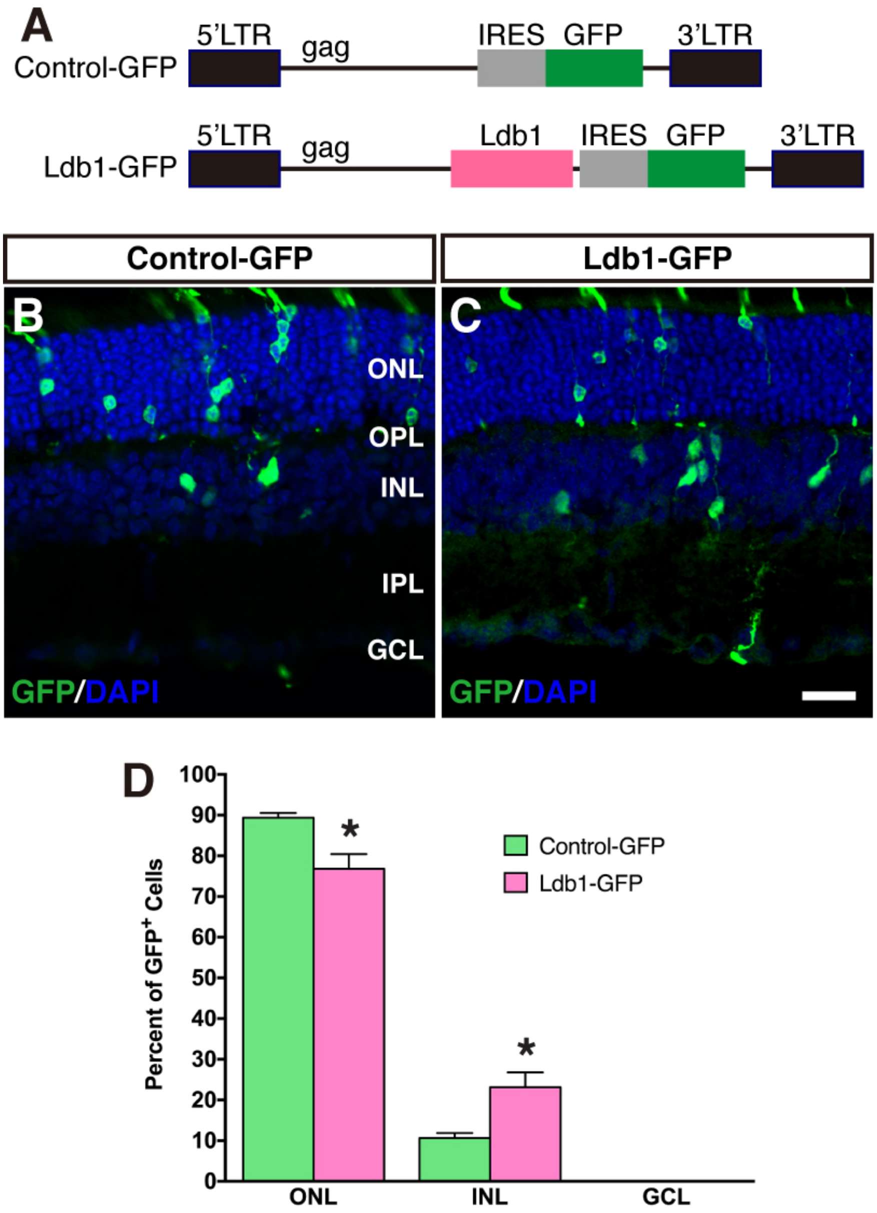

Figure S4 Expression patterns of the Ldb1-GFP and control-GFP retroviruses in the retina. **(A)** A schema to show the structures of the Ldb1-GFP and control-GFP retroviral constructs. **(B,C)** Retroviruses were injected into the subretinal space at P0. Retinal samples were collected at P21 for analysis. The representative retinal sections show that there are relatively more GFP^+ cells (green) migrated into the INL when infected with the Ldb1-GFP viruses. Cells were counterstained with DAPI (blue). **(D)** Quantification of the percentage (means ± SD) of GFP^+ cells within the ONL, INL and GCL in retinas infected with the control-GFP or Ldb1-GFP retroviruses. Three retinas were scored for each virus and more than 1230 GFP^+ cells were counted in each retina. *$p < 0.005$. Abbreviations: GCL, ganglion cell layer; INL, inner nuclear layer; IPL, inner plexiform layer; ONL, outer nuclear layer; and OPL, outer plexiform layer. Scale bar: B,C, 20 μm.

Table S1. Retinal cell types and subtypes immunolabeled by specific antibodies.

Table S2. Gene-specific primer sequences used for qRT-PCR analysis.

Table S3. List of genes differentially expressed between P0 control and *Ldb1* mutant retinas as determined by RNA-seq analysis.
